# Terrorism, Radicalisation, Extremism, Authoritarianism and Fundamentalism: A Systematic Review of the Quality and Psychometric Properties of Assessments

**DOI:** 10.1371/journal.pone.0166947

**Published:** 2016-12-21

**Authors:** Akimi Scarcella, Ruairi Page, Vivek Furtado

**Affiliations:** 1 University of Stirling, Stirling, United Kingdom; 2 Mental Health and Wellbeing, Warwick Medical School, University of Warwick, Coventry, United Kingdom; 3 Birmingham and Solihull Mental Health NHS Foundation Trust, Birmingham, United Kingdom; Yokohama City University, JAPAN

## Abstract

**Background:**

Currently, terrorism and suicide bombing are global psychosocial processes that attracts a growing number of psychological and psychiatric contributions to enhance practical counter-terrorism measures. The present study is a systematic review that explores the methodological quality reporting and the psychometric soundness of the instruments developed to identify risk factors of terrorism, extremism, radicalisation, authoritarianism and fundamentalism.

**Method:**

A systematic search strategy was established to identify instruments and studies developed to screen individuals at risk of committing extremist or terrorist offences using 20 different databases across the fields of law, medicine, psychology, sociology and politics. Information extracted was consolidated into two different tables and a 26-item checklist, reporting respectively background information, the psychometric properties of each tool, and the methodological quality markers of these tools. 37 articles met our criteria, which included a total of 4 instruments to be used operationally by professionals, 17 tools developed as research measures, and 9 inventories that have not been generated from a study.

**Results:**

Just over half of the methodological quality markers required for a transparent methodological description of the instruments were reported. The amount of reported psychological properties was even fewer, with only a third of them available across the different studies. The category presenting the least satisfactory results was that containing the 4 instruments to be used operationally by professionals, which can be explained by the fact that half of them refrained from publishing the major part of their findings and relevant guidelines.

**Conclusions:**

A great number of flaws have been identified through this systematic review. The authors encourage future researchers to be more thorough, comprehensive and transparent in their methodology. They also recommend the creation of a multi-disciplinary joint working group in order to best tackle this growing contemporary problem.

## Introduction

In recent years, there has been an increase in political, religious and ideological violence across the globe. This has resulted in an almost fivefold increase in terrorism-related fatalities since 9/11. According to published figures, the number of fatalities has steadily grown over the last 14 years, from 3,361 in 2000 to 11,133 in 2012 and 17,958 in 2013 [1]. The four terrorist groups mainly responsible for these deaths include Islamic State (IS) in Iraq and Syria, Boko Haram in Nigeria, the Taliban in Afghanistan, and al-Qaida in various parts of the world.

Radicalisation is defined as the process by which an individual or group comes to adopt increasingly extreme political, social, or religious ideals and aspirations that either 1) reject or undermine the status quo or 2) reject or undermine contemporary ideas and expressions of freedom of choice [2]. It differs from extremism, which is defined by the British Government as vocal or active opposition to fundamental values, including democracy, the rule of law, individual liberty, and mutual respect and tolerance of different faiths and beliefs [3]. Home-grown, violent extremists are a current well-recognised concern in UK society, with increasing cases of radicalisation even being picked up in schoolchildren [4] (*see* Table 1 for definitions).

**Table 1:** Definitions of concepts used in the systematic review.

Concept	Definition
Extremism	Vocal or active opposition to fundamental values, including democracy, the rule of law, individual liberty, and mutual respect and tolerance of different faiths and beliefs [3]
Terrorism	The unofficial or unauthorised use of violence and intimidation in the pursuit of political aims [5]
Fundamentalism	Belief that there is one set of religious teachings that clearly contains the fundamental, basic, intrinsic essential, inerrant truth about humanity and deity [6]
Radicalisation	The process by which an individual or group comes to adopt increasingly extreme political, social, or religious ideals and aspirations that either reject or undermine the status quo or reject and/or undermine contemporary ideas and expressions of freedom of choice [2]
Authoritarianism	Unqualified submission to authority, as opposed to individual freedom of thought and action [7]

The Channel Project (2007) was introduced in the UK following the London 7/7 attacks in 2005 with the aim of identifying people who are vulnerable to extremism, to then refer them to the appropriate agencies to address their extremist behaviour and keep them away from danger [4]. Up until 2014, a total of 3,934 UK residents, of all ages, have been referred to Channel since launch in 2006/07, according to figures from the National Police Chiefs Council, with approximately one fifth (777), being assessed as being at risk of radicalisation and referred to receive specialist support [8]. However, in 2015 alone, more than 3,800 UK residents were referred to the programme, with approximately two thirds (2,629) dedicated to ‘Islamic extremism’ referrals. This number, almost as large as that accumulated in the past eight years, illustrates the continuous growth of the programme [9].

There has been a marked increase in referrals to the Channel Project over the last four years, since 2012, with regards to children, with a total 423 children under the age of 18 being referred to the scheme in 2013/14 compared to 290 referrals in 2012/13 [8]. This increase has appeared to have occurred alongside recent events such as the civil war in Syria, and the rise of the Islamic state in Iraq and the Levant (ISIL).

As reported in *“Preventing Religious Radicalisation and Violent Extremism*: *A Systematic Review of the Research Evidence”* [10], there has been a growing body of literature investigating the process of radicalisation, however the majority of available literature is focused on terrorism rather than radicalisation. Evidence regarding radicalisation focuses on violent radicalisation as opposed to non-violent radicalisation, thus introducing a systematic bias in the literature, away from any radicalisation process preceding terrorism but not resulting in acts of violence [10].

Previous reviews have determined that, at present, there is limited evidence base for interventions used in effectively preventing violent extremism [10]. However, this does not address the root of the problem, which is the necessity to identify those individuals who are at risk of being radicalised, so that they can be appropriately signposted for further risk assessment. This brings to the forefront the need for a well evidenced tool of highlighting those individuals that are at risk of radicalisation.

It is only over the last 20 years that valid risk assessment tools have been developed [1]. Forensic psychiatrists and psychologists regularly use risk assessment tools such as the HCR-20 and SAVRY to determine risk of violence in psychiatric patients [1]. These two can be critiqued due to the large populations which they are used with, but scales used to determine low prevalent characteristics such as the risks of extremism/radicalisation, are much more difficult, especially when the population size of interest is low. Therefore, due to the relatively low base rate of extremist and radicalised individuals, it is much more difficult to create empirically based, actuarial prediction instruments to determine the risk of both violent and non-violent extremism/radicalisation [1].

Psychometric scales and assessment tools currently used to measure features of radicalisation, as a psychological construct, include the published Revised Religious Fundamentalism Scale and the Violent Extremist Risk Assessment (VERA-2), and the unpublished Extremist Risk Guidance 22+ (ERG 22+), of which there is very little obtainable information about.

The Revised Religious Fundamentalism Scale aims to measure religious fundamentalism, and is a short research scale of 12 items. The VERA-2, however, is a specialised risk assessment tool that is designed to be used with individuals with either a history of extremist violence, and is applicable only to the cohort that is in an operational phase of violent extremism, for example the population within Tier 4 (those actively breaking the law) of the UK government’s terrorist pyramid [10]. It is designed to be systematic, empirically grounded, developmentally informed, treatment oriented, flexible and practical, and includes factors known to be relevant to the process of radicalization leading to violent extremism [10]. VERA-2 is supported by extensive research undertaken in the area of radicalization and terrorism, in particular in Canada, and is an instrument designed to be used operationally to give a qualified opinion as to which individuals might be at risk for violent extremism [1].

To date, there has not been a published unbiased critical review of all rating scales used to identify individuals at risk related to radicalisation or extremism. It is unclear as to whether the rating scales that are used have in fact been reviewed or evidenced at all. Additionally, the existing work of counter-terrorism strategies undertaken up until now, such as the UK’s government PREVENT, has received a considerable amount of criticism [11]. When trying to implement strategies to combat a problem of this magnitude, legislators must keep an open-mind and implement feasible and reasonable programmes, or else, the only apparent result is that the very own purpose of these strategies is defeated, as it risks to alienate the professionals with whom they seek to work with [12].

This paper systematically evaluates all rating scales used in radicalisation and extremism in order to fill this research gap and to identify areas for future research. The aim of this paper is to solely review current tools used in radicalisation and extremism, without attempting to find an association between mental illness and/or personality traits influencing the risk of radicalisation and extremism.

## Method

Many questionnaires and other tools have been devised in an attempt to identify individuals at risk of participating in terrorist and extremist acts of violence. To date, no research has been conducted to review the strengths and weaknesses of such tools. This systematic review aims to critically appraise questionnaires, rating scales, inventories, and other tools predicting and assessing psychological markers, affinities and attitudes towards terrorism, extremism, radicalisation and ideas conveyed by those concepts. To do so, the specific objectives of this study were to provide an overview of the existing tools developed and their background, to assess their quality, psychometric properties (*see* Table 2), and more specifically their validity and reliability.

**Table 2:** Definitions of psychometric properties.

Psychometric property	Definition
Readability	Ease at which the reader can understand a written text, and depends on content and typography.
Cultural translation	Appropriate translation of the tool so that it can be readily understood and accepted by members of different cultural backgrounds.
Respondent burden	Presumed hardship that is entailed in being a survey participant, for example response fatigue, social stigma etc.
Content validity	Measure inquiring whether the data (content) obtained from the test/rating scale are in line with the general objectives or specifications that the data scale is designed to measure (risk of radicalisation/extremism).
Criterion validity	Ability of the test/rating scale to calculate a result against an external criterion such as another test/rating scale (concurrent validity) or future diagnostic possibility (predictive) of risk of radicalisation/extremism.
Construct validity	Ability of the test/rating scale to individually measure the theoretical construct of interest (an individual’s risk of radicalisation and/or extremism), and is made up of content validity, criterion validity, incremental validity, convergent validity, discriminant validity and experimental validity
Internal consistency	Measure based on the correlations between different items on the same test/rating scale (or the same subscale on a larger test), verifying whether several items on an individual test/scale that propose to measure the same general construct (risk of radicalisation and/or extremism) produce similar scores to each other
Inter-rater reliability	Degree of agreement amongst the raters who complete the rating scale in determining whether the tested individual is at risk of radicalisation/extremism, giving a score of how much homogeneity, or consensus, there is in the ratings given by people completing the test/scale.
Intra-rater reliability	Degree of agreement regarding the tested individual’s risk of radicalisation/extremism amongst repeated administration of the same individual test/rating scale performed by a single rater.
Test-retest reliability	Measure of the degree to which results from a test or rating scale are consistent over time, by delivering the same test to the same individuals on two occasions and collating the scores.
Positive predicted value (PPV)	Probability that subjects with a positive rating score show that they are at risk of radicalisation/extremism truly have this risk, and is calculated as the number of true positives (i.e. correct identification of risk of radicalisation/extremism) divided by the total number of true positives and false positives (false identification of an individual being at risk of radicalisation/extremism when there is no risk present).
Negative predicted value (NPV)	Probability that subjects with a negative rating score for risk of radicalisation/extremism truly don't have this risk and is calculated as the number of true negatives (i.e. true rejection of an individual being at risk of radicalisation/extremism) divided by the total number of true negatives and false negatives (false rejection of an individual being at risk of radicalisation/extremism when this risk is indeed present).
Sensitivity (true positive rate)	Measure of the proportion of positives correctly identified as such. In our study, sensitivity is the percentage of people at risk of radicalisation/extremism who are correctly identified by the test as having this risk.
Specificity (true negative rate)	Measure of the proportion of negatives correctly identified as such. In our study, specificity is the percentage of people who are not at risk of radicalisation/extremism who are correctly identified by the test as not having this risk.
Floor effect	When a lower limit of data value is present that a data collection tool can reliably specify, the lower limit is known as the "floor".
Ceiling effect	Level at which an independent variable (i.e. a risk factor for radicalisation/extremism) no longer has an effect on a dependent variable (risk of an individual becoming “radicalised”), and also refers to the level above which the variance in an independent variable can no longer be measured.
Responsiveness	Ability of a data collection tool to change over a pre-specified time frame, compared to external responsiveness, reflecting the extent to which change in the data collected from the same tool relates to a corresponding change in a reference measure (i.e. threshold for determining risk of radicalisation/extremism).

### Review protocol

In order to ensure a transparent and comprehensive report of results, this study followed the Preferred Reporting Items for Systematic Reviews and Meta-Analyses (PRISMA) statement [13]. Doing so ensured a thorough and accurate reporting of the methodology and results.

### Systematic search strategy

To optimise the systematic literature search, 20 databases were searched across five different subject areas, namely law, medicine, psychology, sociology and politics, and included the following: Westlaw, Index to Legal Periodicals and Books, Index to Foreign Legal Periodicals, HeinOnline, Wiley Online Library, LexisNexis Library, MEDLINE, PubMed, PsychINFO, PsychARTICLES, PsychTESTS, Scopus, Applied Social Science Index and Abstract, International Bibliography of the Social Sciences, Web of Science, Race Relations Abstracts, Sociological Abstracts, International Political Science Abstracts, Worldwide Political Science Abstracts, and Declassified Documents Reference System. After having met with information specialists, the search strategy consisted of a combination of the following Boolean keywords: (extrem* OR radicali* OR terroris* OR salafi* OR islam* OR jihad* OR ideolog* OR *“sleeper cell”* OR *“political violence”* OR *“suicide bombing”*) AND (question* OR screen* OR trait* OR profil* OR assess* OR item* OR characteristic* OR *“risk factor”* OR *scale* OR *criteria*). Due to the extensive list of databases searched, and given the scope of the literature covered in these platforms, further individual searches were developed for each database as to strengthen the quality of the overall search. Comprehensive and detailed information was made available from the authors upon request. No language or time restrictions were applied, resulting mostly in English-language articles, but the search nevertheless included publications in French, Italian, Spanish and German. Articles in different languages were not analysed by the authors due to resource limitations. As an additional quality control standard, and when given this possibility by the database search engines, the search was restricted to peer-reviewed articles only. The databases were last assessed on March 13, 2016. However, a handful of articles have been further assessed following the peer review process.

**Fig 1:**
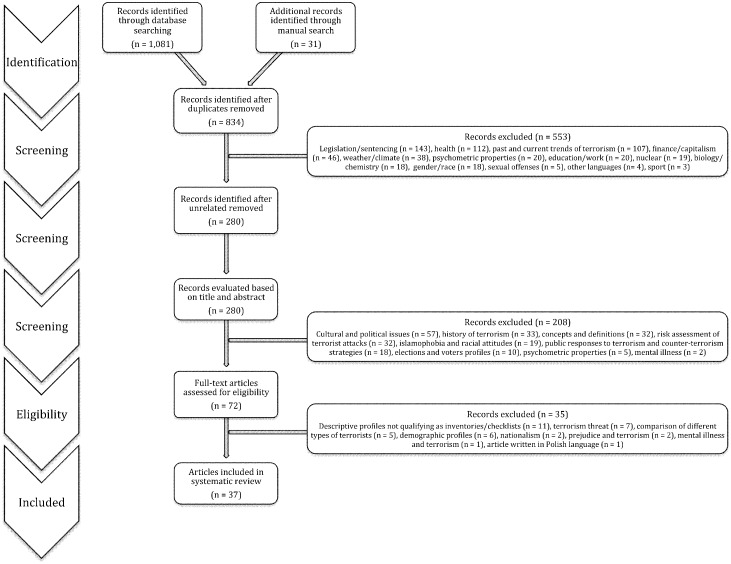
Flow diagram of the systematic search strategy for tools predicting and assessing psychological predictors, affinities, and attitudes towards terrorism, extremism, radicalisation and ideas conveyed by those concepts.

After removal of the duplicates, the initial search revealed a total of 816 records, to which were added manually retrieved articles (n = 31), amounting to a total of 835 records (Fig 1). These additional articles were located through analyses of bibliographies and reference sections of the initial search results.

### Study selection

After having completed the computerised search of all 20 databases, two reviewers independently assessed the records one by one, and identified unrelated titles. Such a strategy enabled the exclusion of 553 records (*see* Fig 1 for detailed reasons of exclusion).

In the second stage of the selection, two reviewers worked independently and assessed the records based on their titles and abstracts. Applying the inclusion and exclusion criteria delineated below, the records deemed not relevant to the question were excluded, and corresponded to a total of 208 records (*see* Fig 1 for detailed reasons of exclusion).

The third and final stage consisted of a full-text screening of the records by both reviewers. Upon assessment, each reviewer independently decided which articles were eligible to be included in the systematic review. Any disagreement was resolved by a discussion between the two reviewers. When the two reviewers were unable to find consensus, a third reviewer was consulted to make the final decision. This final screen yielded the exclusion of 35 records (*see* Fig 1 for detailed reasons of exclusion).

A total of 37 articles were included in the systematic review and consisted of 4 instruments designed to be used operationally by professionals to give an opinion on individuals that might be at risk, 17 tools developed as research measures of a particular and relevant psychological construct, and 9 inventories that have not been generated from a study.

### Inclusion and exclusion criteria

#### Study type and design

Any study using a psychometric tool identifying potential risk factors and/or indicators of individuals likely to engage in or to sympathise with acts of terrorism, radicalisation, extremism, authoritarianism, and/or fundamentalism were included. A wide array of instruments was found, ranging from assessments, questionnaires, surveys, rating scales, guidelines, screenings, inventories, and check-lists. For the sake of clarity, this systematic review adopted a threefold categorisation and classified by instruments to be used operationally by professionals, tools developed as research measures of a particular psychological construct, and inventories not generated from a study. However, the latter category was included in this research purely for information purposes, as they do not hold any empirical ground *per se*, given that they do not derive from studies. The authors believed they were nonetheless worth mentioning, as they could lead to potential future research.

#### Population

No restrictions were applied to the population.

#### Domains assessed

Any study aimed at examining aspects of terrorism, radicalisation, extremism, authoritarianism and/or fundamentalism. It is beyond the scope of this paper to appraise studies attempting to identify risk factors of ‘general’ violence, ‘common’ offenders, nationalist tendencies or Islamophobia, or to identify an association with mental illness. Therefore, any study related to this broader picture was excluded. Likewise, studies that only aimed at establishing demographic profiles of terrorists were excluded.

### Study characteristics, data extraction and quality assessment

A formal threefold classification of the articles depending on the type of tool the studies were making use of was created: instruments to be used operationally by professionals, tools developed as research measures, and inventories not generated from a study.

Each study was thoroughly analysed, and the information was extracted into standardised coding sheets.

Information concerning the details of each study was consolidated in a first coding sheet (*see* Appendix A in S1 File), and included the following characteristics:

1. Country and setting of the study/tool
2. Domain(s) assessed by the tool
3. Population—number of respondents and gender distribution
4. Age of the respondents—age range, mean, and standard deviation
5. Description of items used in the study/tool

An additional coding sheet was developed to extract background information (*see* Table 3):

1. Year of publication
2. Author(s)
3. Area of expertise of the author(s)
4. Journal of publication

**Table 3:** Background summary of studies/tools and abbreviations used in the systematic review.

Acronyms and abbreviations used	Study/tool	Year	Author(s)	Author(s) background	Journal of publication
1992-RWA	Right-Wing Authoritarianism Scale	1992	B. Altemeyer, B. Hunsberger	Psychology	The International Journal for the Psychology of Religion
Borum (2014)	Borum’s propensities for involvement with violent extremism	2014	R. Borum	Psychology	Behavioral Sciences and the Law
ARIS	Activism and Radicalism Intention Scale	2009	S. Moskalenko, C. McCauley	Homeland Security, Psychology	Terrorism and Political Violence
ARIS-S	Activism and Radicalism Intention Scale—Spanish Version	2016	H.M. Trujillo, M. Prados, M. Moyano	Psychology, Philosophy	International Journal of Social Psychology
EMI-20	Extremism Monitoring Instrument	2014	A.P. Schmid	Terrorism Research	ICCT Research Paper
ERG 22+	Extremism Risk Guidelines	2011	National Offender Management Service	Psychology	Journal of Threat Assessment and Management
ERS	Extremism Risk Screen	2011	National Offender Management Service	Psychology	Journal of Threat Assessment and Management
Horgan (2008)	Horgan’s predisposing risk factors for involvement in terrorism	2008	J. Horgan	Psychology	The ANNALS of the American Academy of Political and Social Science
IFS	Islamic Fundamentalism Scale	2014	I.E. Putra, Z.A. Sukabdi	Psychology	Peace and Conflict: Journal of Peace Psychology
ITFS	Intra-Textual Fundamentalism Scale	2007	W.P. Williamson, A. Ahmad	Psychology	Journal of Muslim Mental Health
IVPG (study A)	Identifying Vulnerable People Guidance	2015	J. Cole, E. Alison, L. Alison	Psychology	PREVENT guidance document
IVPG (study B)	Identifying Vulnerable People Guidance	2016	V. Egan, J. Cole, S. Elntib	Psychology	Journal of Threat Assessment and Management
Kebbell & Porter (2012)	Risk factors associated with violent extremism	2012	M.R. Kebbell, L. Porter	Psychology	Security Journal
MDFI	Multi-Dimensional Fundamentalism Inventory	2011	J. Liht, L.G. Conway III, K. O’Neill	Psychology	Archive for the Psychology of Religion
MEMS	Militant Extremist Mind-Set	2010	L. Stankov, G. Saucier, G. Knezevic	Pedagogy and Practice, Psychology	Psychological Assessment
MMPI-2	Minnesota Multiphasic Personality Inventory	2004	M. Gottschalk, S. Gottschalk	Psychology	The American Sociologist

A preliminary and methodological quality check was conducted using the 26-item checklist developed by Bennet and colleagues (2011), and constituted the third coding sheet of this systematic review (*see* Table 4).

**Table 4:** Methodological quality markers in the different articles analysed.

Study/tool	Background				Methods			Sample selection			Research tool			Results			Response rates			Interpretation & discussion				Ethics & disclosure		
	Justification of research methods	Background literature review	Explicit research questions	Clear study objectives	Description of methods used	Method of administration	Number and types of contact	Sample size calculation	Representativeness	Method of sample calculation	Description of the research tool	Instrument pretesting	Instrument reliability & validity	Results of research presented	Results address objectives	Generalizability	Response rate stated	How response rate was calculated	Discussion of non-response rate bias	Interpret and discuss findings	Conclusion	Recommendations	Limitations	Consent	Sponsorship	Research ethics approval
**Assessments used operationally by professionals**																										
VERA-2 (study A)	**+**	**+**	-	**+**	**+**	-	-	-	-	-	**+**	-	-	**+**	**+**	-	-	-	-	**+**	**+**	-	**+**	-	-	-
VERA-2 (study B)	**+**	**+**	**+**	**+**	**+**	-	-	**+**	-	-	**+**	-	**+**	**+**	**+**	-	-	-	-	**+**	**+**	**+**	**+**	-	-	-
ERG 22+	**+**	**+**	**+**	**+**	**+**	**+**	-	-	-	-	**+**	-	-	**+**	**+**	-	-	-	-	**+**	**+**	-	**+**	**+**	-	-
ERS	-	-	-	-	-	-	-	-	-	-	-	-	-	-	-	-	-	-	-	-	-	-	-	-	-	-
IVPG (study A)	**+**	**+**	-	**+**	**+**	-	-	-	-	-	**+**	-	-	**+**	**+**	-	-	-	-	**+**	**+**	-	-	-	-	-
IVPG (study B)	**+**	**+**	-	**+**	**+**	**+**	-	**+**	**+**	-	**+**	-	**+**	**+**	**+**	-	-	-	-	**+**	**+**	**+**	**+**	-	**+**	-
**Tools developed as research measures**																										
1992-RWA	**+**	**+**	-	-	**+**	-	-	**+**	**+**	-	**+**	-	**+**	**+**	**+**	-	-	-	-	**+**	-	-	-	-	-	-
RF-R	**+**	**+**	-	-	**+**	**+**	**+**	**+**	-	-	**+**	**+**	**+**	**+**	**+**	**+**	-	-	-	**+**	-	-	-	-	-	-
PHS	**+**	**+**	**+**	**+**	**+**	-	-	**+**	**+**	-	**+**	-	-	**+**	**+**	-	**+**	-	-	**+**	**+**	-	-	-	-	-
MMPI-2	**+**	**+**	**+**	**+**	**+**	-	-	**+**	**+**	-	**+**	-	-	**+**	**+**	-	**+**	-	-	**+**	**+**	-	-	-	-	-
RWA-R	**+**	**+**	**+**	**+**	**+**	**+**	**+**	**+**	**+**	-	**+**	**+**	**+**	**+**	**+**	**+**	-	-	-	**+**	**+**	-	-	**+**	-	-
ITFS	**+**	**+**	-	**+**	**+**	**+**	**+**	**+**	**+**	-	**+**	**+**	**+**	**+**	**+**	-	**+**	-	**+**	**+**	-	-	-	-	-	-
ARIS	**+**	**+**	**+**	**+**	**+**	**+**	-	**+**	**+**	**+**	**+**	**+**	**+**	**+**	**+**	**+**	**+**	-	-	**+**	**+**	-	-	-	-	-
NBMASA	**+**	**+**	**+**	**+**	**+**	**+**	**+**	**+**	**+**	**+**	**+**	-	**+**	**+**	**+**	-	**+**	-	-	**+**	-	-	-	**+**	**+**	**+**
MEMS	**+**	**+**	**+**	**+**	**+**	**+**	-	**+**	**+**	**+**	**+**	-	**+**	**+**	**+**	-	-	-	-	**+**	-	-	-	-	-	-
MDFI	**+**	**+**	**+**	**+**	**+**	**+**	**+**	**+**	-	**+**	**+**	-	**+**	**+**	**+**	**+**	-	-	-	**+**	-	-	**+**	**+**	-	-
RF-I	**+**	**+**	-	**+**	**+**	**+**	**+**	**+**	**+**	-	**+**	-	**+**	**+**	**+**	-	-	-	-	**+**	-	-	-	**+**	-	-
SyfoR	**+**	**+**	**+**	**+**	**+**	**+**	**+**	**+**	**+**	**+**	**+**	-	**+**	**+**	**+**	**+**	-	-	-	**+**	-	**+**	-	**+**	-	**+**
IFS	**+**	**+**	**+**	**+**	**+**	-	-	**+**	**+**	**+**	**+**	-	**+**	**+**	**+**	-	-	-	-	**+**	**+**	-	-	-	-	-
SSS (studies A-B)	**+**	**+**	**+**	**+**	**+**	**+**	**+**	**+**	**+**	-	**+**	**+**	**+**	**+**	**+**	**+**	**+**	**+**	-	**+**	**+**	**+**	**+**	**+**	-	-
ARIS-S	**+**	**+**	**+**	**+**	**+**	-	-	**+**	**+**	**+**	**+**	**+**	**+**	**+**	**+**	-	-	-	-	**+**	-	-	-	-	-	-
Schbley (2003)	**+**	**+**	**+**	**+**	**+**	**+**	**+**	**+**	**+**	-	**+**	-	**+**	**+**	**+**	-	**+**	-	-	**+**	**+**	**+**	**+**	-	**+**	-
TCS	**+**	**+**	-	**+**	**+**	**+**	-	**+**	**+**	-	**+**	-	**+**	**+**	**+**	-	**+**	-	-	**+**	-	**+**	**+**	-	-	-
TRAP-18	**+**	**+**	**+**	**+**	**+**	**+**	-	-	-	-	**+**	**+**	**+**	**+**	**+**	**+**	-	-	-	**+**	**+**	**+**	**+**	-	-	-

**+**, characteristic present in study;

- characteristic not present in study.

Finally, the authors developed a fourth and final coding sheet to explore the psychometric properties of each tool, and hence assess their validity and reliability (*see* Table 5). The extracted properties (*see* Table 2 for the definitions) included:

1. Readability, cultural translation, respondent burden
2. Validity (content, criterion, construct)
3. Internal consistency
4. Reliability (inter-rater, intra-rater, test-retest)
5. Positive and negative predicted value
6. Sensitivity and specificity
7. Floor and ceiling effect
8. Responsiveness

**Table 5:** Studies/tools psychometric properties.

Study/tool	Readability	Cultural translation	Respondent burden	Content validity	Criterion validity	Construct validity	Internal consistency	Inter-rater reliability	Intra-tater reliability	Test-retest reliability	Positive predicted value	Negative predicted value	Sensitivity	Specificity	Floor effect	Ceiling effect	Responsiveness
**Instruments used operationally by professionals**																	
VERA-2 (study A)	**++**	**+**	**++**	**++**	**–**	**–**	**–**	**–**	**–**	**–**	**–**	**–**	**–**	**–**	**–**	**–**	**–**
VERA-2 (study B)	**++**	**+**	**++**	**++**	**–**	**–**	**–**	**++**	**–**	**–**	**–**	**–**	**–**	**–**	**–**	**–**	**–**
ERG 22+	**++**	**+**	**++**	**++**	**–**	**–**	**–**	**–**	**–**	**–**	**–**	**–**	**–**	**–**	**–**	**–**	**–**
ERS	**–**	**–**	**–**	**–**	**–**	**–**	**–**	**–**	**–**	**–**	**–**	**–**	**–**	**–**	**–**	**–**	**–**
IVPG (study A)	**++**	**–**	**++**	**–**	**–**	**–**	**–**	**–**	**–**	**–**	**–**	**–**	**–**	**–**	**–**	**–**	**–**
IVPG (study B)	**++**	**–**	**++**	**–**	**–**	**–**	**+**	**+**	**–**	**–**	**+**	**++**	**+**	**+**	**–**	**–**	**–**
**Tools developed as research measures**																	
1992-RWA	**+**	**+**	**++**	**–**	**–**	**++**	**++**	**–**	**–**	**–**	**–**	**–**	**–**	**–**	**–**	**–**	**–**
RF-R	**++**	**+**	**++**	**–**	**–**	**++**	**++**	**–**	**–**	**–**	**–**	**–**	**–**	**–**	**–**	**–**	**–**
PHS	**–**	**–**	**+**	**–**	**–**	**–**	**–**	**–**	**–**	**–**	**–**	**–**	**–**	**–**	**–**	**–**	**–**
MMPI-2	**++**	**–**	**+**	**+**	**+**	**+**	**–**	**–**	**–**	**–**	**–**	**–**	**–**	**–**	**–**	**–**	**–**
RWA-R	**++**	**+**	**++**	**–**	**–**	**++**	**+**	**–**	**–**	**–**	**–**	**–**	**–**	**–**	**–**	**–**	**–**
ITFS	**++**	**–**	**++**	**+**	**+**	**+**	**–**	**–**	**–**	**–**	**–**	**–**	**–**	**–**	**–**	**–**	**–**
ARIS	**++**	**+**	**++**	**–**	**–**	**++**	**++**	**–**	**–**	**–**	**–**	**–**	**–**	**–**	**–**	**–**	**–**
NBMASA	**++**	**++**	**++**	**–**	**–**	**++**	**++**	**–**	**–**	**–**	**–**	**–**	**–**	**–**	**–**	**–**	**–**
MEMS	**++**	**++**	**++**	**–**	**–**	**++**	**+**	**–**	**–**	**–**	**–**	**–**	**–**	**–**	**–**	**–**	**–**
MDFI	**++**	**++**	**++**	**–**	**++**	**++**	**++**	**++**	**–**	**–**	**–**	**–**	**–**	**–**	**–**	**–**	**–**
RF-I	**++**	**++**	**++**	**–**	**–**	**++**	**+**	**–**	**–**	**–**	**–**	**–**	**–**	**–**	**–**	**–**	**–**
SyfoR	**++**	**+**	**++**	**++**	**++**	**++**	**++**	**–**	**–**	**–**	**–**	**–**	**–**	**–**	**–**	**–**	**–**
IFS	**+**	**–**	**++**	**–**	**–**	**–**	**+**	**–**	**–**	**–**	**–**	**–**	**–**	**–**	**–**	**–**	**–**
SSS (studies A-B)	**++**	**++**	**++**	**–**	**–**	**++**	**+**	**–**	**–**	**–**	**–**	**–**	**–**	**–**	**–**	**–**	**–**
ARIS-S	**++**	**++**	**++**	**–**	**–**	**++**	**++**	**–**	**–**	**–**	**–**	**–**	**–**	**–**	**–**	**–**	**–**
Schbley (2003)	**+**	**+**	**++**	**++**	**++**	**–**	**+**	**–**	**–**	**–**	**–**	**–**	**–**	**–**	**–**	**–**	**–**
TCS	**++**	**+**	**+**	**++**	**–**	**–**	**++**	**–**	**–**	**–**	**–**	**–**	**–**	**–**	**–**	**–**	**–**
TRAP-18	**++**	**+**	**++**	**++**	**–**	**–**	**–**	**++**	**–**	**–**	**–**	**–**	**+**	**–**	**–**	**–**	**–**

**–**, no information available.

Readability: **+**, items available but lengthy; **++**, items available, short and comprehensive. Cultural translation: **+**, only available in English; **++**, available in English and also language(s) of the target population. Respondent burden: **+**, over 60 items; **++**, under 60 items. Content validity: **++**, experts consulted. Criterion validity: **++**, correlation coefficients calculated (Spearman’s, Pearson’s Kendall’s and/or Cramer’s). Construct validity: **++**, factor analysis conducted. Internal consistency: **+**, mean Cronbach’s alpha inferior to .80; **++**, mean Cronbach’s alpha superior or equal to .80. Inter-rater validity: **+**, mean Cohen’s kappa inferior to .70; **++**, mean Cohen’s kappa superior or equal to .70. Intra-rater validity: **+**, moderate; **++**, satisfactory. Positive predicted value: **+**, moderate; **++**, satisfactory. Negative predicted value: **+**, moderate; **++**, satisfactory. Sensitivity: **+**, moderate; **++**, satisfactory. Specificity: **+**, moderate; **++**, satisfactory.

This process was conducted by two reviewers, with the second reviewer completing a proportion for quality control. Where necessary, any disagreement was resolved by discussion; if this failed, the third reviewer made a final decision.

### Methods of review

Following the threefold division developed by the authors, this systematic review will discuss the background information of the selected tools. A primary assessment of the methodological quality markers of the studies will then be conducted, after which will ensue an in-depth analysis of their psychometric properties, and overall validity and reliability. Finally, preliminary conclusions will be drawn.

### Data synthesis

As delineated above, the findings were synthesised in clear and concise tables in order to smooth comparisons, and were subsequently reported in a narrative overview.

## Results

### Overall description of the studies and tools

A total of 37 articles were included and reviewed using the PRISMA tool (*see* Fig 1 and S1 File for details). This analysis resulted in a total of 6,636 respondents between the ages of 14 and 92 years, encompassing all geographical continents with Belarus, Italy, Serbia, Slovakia, Spain and the U.K. representing Europe; China, Korea, Kyrgyzstan, Malaysia, Pakistan and Sri Lanka representing Asia; Canada, Guatemala, Mexico and the U.S.A. representing North America; Israel and Lebanon representing the Middle East; Chile representing South America; Australia representing Oceania; and one study indicated sampling African respondents. In addition to geographical coverage, the respondents came from various backgrounds. Some sampled high school and college students and their parents, others were directed at Christian and Muslim beliefs, whereas other studies sampled volunteers in places of worship or members or religious seminaries. Certain studies included samples of sympathisers, activists and militants of radical movements as well, or even incarcerated individuals convicted of terrorist offenses.

The 37 articles were transformed into 30 tools due to the fact that certain articles developed more than one tool at a time, whereas other articles served to analyse the same tool from different perspectives. Of the 30 tools extracted, 12 evaluated attitudes and risk factors of extremism (38%), 8 evaluated terrorism (26%), 5 evaluated fundamentalism (16%), 3 evaluated radicalisation (10%), and finally 2 evaluated authoritarianism (8%).

The tools were separated according to a threefold division. Instruments to be used operationally by professionals included the VERA-2 with two distinct studies [14, 15], ERG 22+ [16], ERS [16] and IVPG with two distinct studies [17]. The tools developed as research measures of particular constructs were the 1992-RWA [6], RF-R [18], PHS [19], MMPI-2 [19], RWA-R [20], ITFS [21], ARIS [22], NBMASA [23], MEMS [24], MDFI [25], RF-I [26], SyfoR [27], IFS [28], SSS [29], ARIS-S [30], TCS [31], TRAP-18 [32] and a tool created by Schbley [33]. Finally, inventories not generated from a study comprised tools developed by Ross [34], Vaisman-Tzachor [35], Horgan [36], Saucier et al. [37], Kebbell and Porter [38], Monahan [39], USAID [40], Borum [41] and the EMI-20 [42].

It must be noted that the last category, inventories not generated from a study, has not been screened for markers of reporting quality, because the checklist developed by Bennett and colleagues is aimed at studies in which information on a specific topic is gathered from a population sample [43], and therefore would not be suitable for other articles. Their checklist was developed in an attempt to appraise the optimal reporting guidelines of survey research. As such, without an empirical study around which the article can revolves, it becomes impossible to successfully identify key reporting domains. Likewise, this review did not analyse the psychometric properties of these inventories, for the simple reason that it is inappropriate for articles that have not tested the tools. Their inclusion in this systematic review was solely aimed at giving a numerical representation of how many instruments are out there awaiting testing, and to stimulate further research in the field.

### Preliminary quality reporting

#### General comments on methodological quality markers

Instruments designed to be used operationally by professionals to take decisions as to people that might be at risk and tools developed as research measures of particular psychological constructs were assessed for markers of reporting quality (*see* Table 4). The first criterion of each section was present in almost all of the studies, but the ensuing ones were incomplete. Those criteria are respectively the *description of methods used for data analysis*, *sample size calculation*, *description of the research tool*, and *interpret and discuss the findings*. The average tool met just over half of the criteria (M = 14.42 of the 26; 55%). All three categories presented satisfactory results for the *background* (M = 3.42 of 4 criteria met) and *results* section (M = 2.94 of 3 criteria), and considerably mediocre results for the *response rates* (0.50 of 3 criteria), and for *ethics and disclosure* (M = 0.67 of 3 criteria). However, the other sections presented mixed results; *research tool* (M = 2.67 of 3 criteria met), *methods* (M = 2.61 of 3 criteria), *sample selection* (M = 2.33 of 3 criteria), *interpretation and discussion* (M = 2.89 of 4 criteria met). In sum, it can be concluded that not a single category, and more specifically, not a single instrument, had a sufficient and satisfactory amount of criteria necessary for a transparent report.

#### Instruments to be used proactively by professionals

Surprisingly, the average instrument to be used operationally by professionals had considerably less developed results than the average tool (M = 10.34 of 26 criteria; 40%). All sections were complete to a lesser extent than the average tool sections, at the exception of the *interpretation and discussion* one (M = 2.67 of 4 criteria): *background* (M = 2.83 of 4 criteria), *methods* (M = 1.17 of 3 criteria), *sample selection* (M = 0.5 of 3 criteria), *research tool* (M = 1.17 of 3 criteria), *results* (M = 1.67 of 3 criteria), *response rates* (M = 0 of 3 criteria), *ethics and disclosure* (M = 0.34 of 3 criteria). However, even the above average results proved to be far insufficient for a transparent description. This shrinkage of scores could somewhat be explained due to the fact that no information was disclosed concerning the ERS. Additionally, one could argue that those results must be interpreted cautiously, as the professional assessments category only consists of 6 studies. Yet, bearing in mind that those assessments are the instruments used by professionals in practice, one cannot help but wonder why studies of such a magnitude have not been conducted in a more thorough and extensive manner.

#### Tools developed as research measures

The opposite phenomenon was observed with the average instrument developed as research measure when compared to the average tool, as it not only scored generally higher (M = 15.72 of 26 criteria; 60%), but scored higher in every separate section as well, if it was not for the *interpretation and discussion* one (M = 2 of 4 criteria). The scores for each section were as follows: *background* (M = 3.61 of 4 criteria), *results* (M = 2.39 of 3 criteria), *research tool* (M = 2.28 of 3 criteria), *methods* (M = 2.22 of 3 criteria), *sample selection* (M = 2.17 of 3 criteria), *ethics and disclosure* (M = 0.56 of 3 criteria), *response rates* (M = 0.50 of 3 criteria). Leaving out the last two, as they were neglected across all studies, and hence do not offer a comprehensive comparison, the studies developing tools as research measures were found to have been constructed with a greater attention to detail, meeting criteria in a more developed way than the other categories. Nonetheless, it is worth noting that even if scoring generally higher than the other categories, the average tool developed as a research measure is found to report less than two thirds of the criteria necessary for a transparent analysis, and are hence still considered poor.

### Validity, reliability, and other psychometric properties

#### General comments on the psychometric properties

After having assessed the general quality markers of all instruments issued from studies, this systematic review further analysed these by extracting their psychometric properties (*see* Table 5). A total of 17 characteristics were considered in order to best assess the validity and reliability of each (*see* Table 2). To the surprise of the authors, the average tool reported less than a third of the 17 selected properties (M = 4.70; 28%), with a report range of only 0 to 8, meaning that the instrument reporting the most properties had only 47% of them. Certain properties were consistently covered by the majority of the tools; *respondent burden* (23 out of 24 studies), *readability* (22 out of 24), *cultural translation* (15 out of 24), *construct validity* (13 out of 24), and *internal consistency* (13 out of 24). However, equally valuable properties were omitted, including *content validity* (only present in 9 out of 24 studies; just over a third), *criterion validity* (only present in 4; 17%), *inter-rater reliability* (only present in 4; 17%), *positive and negative predicted values* (only present in 1; 4%), *sensitivity and specificity* (only present in 1; 4%). Finally, a number of the properties were overlooked by *all* studies; *intra-rater reliability*, *test-retest reliability*, *floor and ceiling effects*, and *responsiveness*.

#### Instruments to be used proactively by professionals

Similar to the preliminary quality screen, the instruments responding to the smallest amount of criteria are the instruments designed to be used by professionals to take decisions as to the potential individuals at risk. The two studies on VERA-2 collected a total of 5, whereas the ERG 22+ amassed 4 out of the 17, hence both reporting only under a third of the properties. The best results were held by the IVPG, with a total of 8, albeit modest in comparison to those of the other tools. Despite the lack of readily available information, the obtained results of the instruments proved rather satisfactory, with almost twice as many upper markings (M = 2.5) than moderate ones (M = 1.33). However, none of the studies reported the internal consistency of their tools, with the exception of the IVPG scoring a modest cronbach’s alpha of .64.

This result is somewhat surprising, as the vast majority of instruments in the other two categories not only discussed that property, but also obtained an alpha superior to .80. Similarly, to the quality markers results, this category proves to be undoubtedly insufficient with regards to the quality of the psychometric properties reported. However, as previously stated, it is advised to take these findings with caution, as the category was composed of only 6 studies.

#### Tools developed as research measures

The second category consistently reported a larger number of psychometric properties (M = 5.00; 28%), although these still amounted to just less than a third of the extracted characteristics. The reported properties were more developed than the instruments used operationally by professionals, with over twice as many upper markings (M = 3.50) than moderate ones (M = 1.50). Most of the questionnaires reported 5 properties, whereas the IFS reported 3, and the PHS reported only one. The MDFI and the SyfoR were the only instruments reporting slightly over a third of the properties, with a total of 7 characteristics discussed. On the other hand, unlike the professional instruments, 14 studies calculated the internal consistency using cronbach’s alpha; 8 of which scored over .80. It is once again worth noting that even if these tools prove to be of a better quality than the instruments designed for professional use, the reporting rate of their psychometric properties still does not exceed 28%.

## Discussion

The aim of this systematic review was to explore the empirical literature evaluating extremism, terrorism, radicalisation, authoritarianism, and fundamentalism in order to extract the developed instruments identifying potential risk factors and/or indicators of individuals likely to engage in or to sympathise with acts of that kind. To our knowledge, this systematic review is the first study presenting a comprehensive and thorough analysis of current tools used to elicit these traits and attitudes in individuals.

To provide a clear and accurate overview of the existing multiple studies, the search strategy followed the PRISMA tool. A search of twenty databases was conducted, and encompassed different domains, namely law, psychology, medicine, sociology, and politics. This yielded a total of 37 peer-reviewed articles, including 4 instruments to be used operationally by professionals, 17 tools developed as research measures of particular constructs, and 9 inventories that have not been generated from studies. Each article focused on measuring different specific aspects, but all were connected to the identification of terrorism, extremism, fundamentalism, radicalisation, and authoritarianism.

Whilst reporting the results, the authors created a checklist to report the psychometric properties of each study, whereas a preliminary quality reporting was conducted using the guidelines established by Bennett and colleagues. The latter is not as exhaustive and as widely recognised in comparison to the PRISMA tool, but rather embodies a primary set of considerations for researchers.

Following inspection of the authors and their background, we further identified that an overwhelming majority of tools (24 of the 33 studies, or 73%), were developed by experts in psychology and other behavioural sciences. Less than a quarter included authors with an expertise in law, criminal justice, and/or homeland security. Finally, only one single tool, the SyfoR, included someone with a medical background among its authors. Due to the multi-faceted nature of terrorism and extremism, one could wonder whether psychologists or legal experts alone are those best equipped to discuss the very nature of this problem.

There were two main findings of this systematic review. First, just over half of the criteria necessary for a transparent description of the instruments were reported across the different studies, and even less were reported in the articles that developed the professional instruments. Second, the same was found for the assessment of the psychometric properties, which was of poor quality overall. It is important to note that the instruments used by experts, and approved by their respective governments, are based on either minimal information or on un-critiqued information, which remains inaccessible to researchers to develop further.

### Implications

The non-existence of any uniform coding of the tools primarily hampered the comparison across studies, hence rendering the assessment of instruments somewhat less comprehensive, less transparent, and, as a result, less valid. However, having valid instruments is crucial in forensic contexts, and even more so in meta-analyses, as substantiated by studies by Hurducas and colleagues [44], White, Meares, and Batchelor [45] and Fusar-Poli and colleagues [46]. Hence, this systematic review argues that the relatively modest performance of all included studies can be attributed to the fact that none of them reported their results according to standardised reporting guidelines. To date, numerous guidelines and checklists have been developed across different fields in order to maximise the assessment of validity and reliability, and the authors strongly encourage researchers to accept such a ‘gold standard’ approach in their upcoming work, especially when those instruments are aimed at professionals to use proactively to identify individuals at risk.

Despite the obvious lack of transparency, and lack of information with regards to the validity and reliability of the tools, the studies varied in the number and type of potential risk factors tested. Most studies focused on extremism and terrorism, but also included an array of tools assessing fundamentalism, radicalisation and authoritarianism. They assessed various predictors ranging from inner beliefs, (socio-) psychological traits and processes acquired, attitudes and tendencies, willingness to engage in extreme means, hostility, mental wellbeing, and other predisposing vulnerabilities. However, on scrutiny of the background of studies, it became evident that certain predictors were undoubtedly study-specific, as the studies were based on peculiar events rather than being created for preventing a particular issue at hand. As a matter of fact, a large number of studies have been created following the 9/11 events. Studies of this nature, even when producing valuable results, are difficult to generalise. This level of precision renders it unclear as to whether or not health-care professionals across different legal jurisdictions could utilise these as a predictive indicator in their clinical practice.

Finally, one could ponder about the ethicality of implementing and proactively using certain instruments and policies without prior disclosure of the study results they were based on. Out of the four professional instruments, the ERG 22+ and ERS have restricted access to *trained* forensic psychologists or probation officers, on the hypothesis that disseminating their guidelines to the general public would be detrimental to their use [16]. As before mentioned, instruments need to be tested, validity and reliability need to be cross-verified, and studies need to be capable of being replicated and critiqued. The practice of withholding transparency of the results obtained prevents the (re-) evaluation of the methodology, data collection and further analyses by other professionals [47]. Hence, basing policies on such limited peer-reviewed information could be detrimental to those affected by it. Historically, it has been proven that empirical studies are at risk of being faulty, and failure to cross-check their validity has harmful consequences [48]. However, defective screening of individuals at risk of committing terrorist or extremist offenses would have different outcomes, as it would ‘merely’ result in the false incrimination of suspects. And similarly, having valid and widely recognised instruments would facilitate the taking into custody of individuals at risk, and hence avoid any potential false negatives situations, where high-potential individuals could mistakenly be released following an insufficient assessment.

To add to the controversy, if the authors of an instrument succeed in identifying all the relevant risk factors, it is their moral duty to share their findings with the wider community of researchers and authorities in order to try and prevent other crimes of this magnitude. However, the authors of the ERG 22+ claimed their tool to be unique and substantially different from the VERA-2. The former adopts a case-formulation approach, in which the presence of a factor combined with its role in the offense is assessed, whereas the latter is merely a conceptual formulation based on literature only [16]. As such, the ERG 22+ is purportedly aimed at guiding practice as well as stimulating further work in the field. The authors of this systematic review nonetheless argue that it is somewhat idealistic to expect results without having previously publicised the primary results.

### Limitations

Whilst this systematic review is the most robust study to date, some limitations should be acknowledged. First of all, due to the vast scope of the literature covered, our study was restricted. The exclusion criteria were precise, and the authors took the liberty to eliminate articles related to ‘general’ violence, nationalism, and other far-reaching topics at the outset. Only studies that specifically assessed extremism, terrorism, fundamentalism, radicalisation, and/or authoritarianism were considered. Dissertations and other unpublished studies were not included, as the systematic review focused solely on peer-reviewed articles. This highlights acceptance of minor publication bias in the review. Another limitation, with respect to publication bias, concerns the range of languages of the articles assessed. The search strategy only covered articles written in English, French, Italian, Spanish, German and Dutch. The search resulted in only five articles written in a different language (two in Arabic, one in Japanese, one in Polish, and one in Lithuanian), but these were excluded due to a lack of resources. Finally, one must bear in mind that, even though all necessary measures were taken to ensure a comprehensive study, the possibility of missing eligible articles can never entirely be excluded.

### Recommendations

Future research is needed to find a pragmatic way to progress efforts in the identification of risk factors of terrorism, extremism, radicalisation, authoritarianism, and fundamentalism, and to provide definitive conclusions about the relative value of the risk factors and attitudes identified by the different measures used.

First and foremost, the creation of joint working groups to review the findings of the articles might add substantial benefit in identifying tangible results in the field. As previously stated, practitioners from the fields of psychology or law alone are not best equipped to develop these instruments. Therefore, commissioning a multi-disciplinary committee to expand on specific research of this issue would be a step in the right direction. Ideally, the working group would include members of different faculties, in order to ensure the generalizability of the results. Reiterating what has been discussed before, this work would subsequently need to be field-tested to ensure valid and reliable results. However, given the recent course of events with an increase in terrorism, which is continuously spreading to new parts of the world, this would need to be fast-tracked in order to have a real and valuable input.

In the attempt to achieve this, researchers are encouraged to use standardised reporting guidelines to ensure a maximal level of transparency and comprehensiveness, and to ensure that the instruments proposed are valid and reliable. Not doing so would amount to research misconduct, and lead to refusal of being published by journal editors [49].

Finally, further research should aim at exploring the potential confounds of previous studies. As previously discussed, most studies have been created following specific acts of terrorism, and hence the possibility that confounds exist is a significant and non-negligible risk.

### Conclusion

In summary, this systematic review highlights, in no uncertain terms, that the methodological reporting and overall quality, together with the psychometric soundness of the identified instruments, are weak and leave room for improvement. Numerous methodological flaws have been identified in all of the studies included, resulting in a limited interpretation and generalisation of the findings they presented. Even though assessments used by professionals are generally assumed to be the gold standard, there is, in this case, limited choice between the evaluated instruments, in that they are all relatively narrow and disclose very little information, if any at all. Based on the quality reporting and on the psychometric properties (or the lack thereof), there is no substantial evidence that would enable the authors to recommend one instrument over another.

The systematic review observed that significant policies and instruments have been, and continue to be developed based on limited information. This review encourage such studies to be published in their entirety and critiqued in order to ensure transparency.

Finally, it is recommended that a multi-disciplinary working committee is established to find a way to help identify individuals at risk of participating in terrorist and extremist acts of violence in a fully comprehensive and evidence-based manner.

## Supporting Information

S1 File Appendix A—Studies and Tools Characteristics. (DOCX)

S2 File PRISMA Checklist. (DOC)
