# Supplementary material for: Terrorism, Radicalisation, Extremism, Authoritarianism and Fundamentalism: A Systematic Review of the Quality and Psychometric Properties of Assessments
Source: PLoS One. 2016 Dec 21;11(12):e0166947. doi: 10.1371/journal.pone.0166947 (PMC5176288; doi:10.1371/journal.pone.0166947)
Supplement: S1 File — (DOCX) [file pone.0166947.s001.docx]

**S1 File - Appendix A** Studies and tools characteristics

| **Study/tool** | | **Country & setting** | **Domain(s) assessed** | **Population** | **Age (years)** | **Items** |
| --- | --- | --- | --- | --- | --- | --- |
| **Instruments used operationally by professionals** | | | | | |  |
| VERA-2 (study A) | | Canada | Violent political extremism | NA – tool developed following feedback from experts working in law enforcement, corrections, and forensic psychology, and has not been issued following a study | | 31 (7 beliefs and attitudes, 7 context and intent, 6 history and capability, 5 commitment and motivation, 6 protective)  3-point scale (low, medium, high) |
| VERA-2 (study B) | | U.K.  Convicted terrorists | Violent political extremism | N = 5  100% male (5) | Range, M and SD not stated | 31 (7 beliefs and attitudes, 7 context and intent, 6 history and capability, 5 commitment and motivation, 6 protective)  3-point scale (low, medium, high) |
| ERG 22+ | | U.K.  Convicted terrorists | Potential to commit extremist offences | N = over 150  No specific number stated  Gender distribution not stated | Range, M and SD not stated | 23 (13 engagement, 6 intent, 3 capability, any additional ‘+’ factor that appears relevant) |
| ERS | | U.K. | Possible interest in extremist groups, causes, or ideas | Not stated | Range, M and SD not stated | Shortened version of the ERG 22+ but items not accessible |
| IVPG (study A) | | U.K. | Violent extremism | NA – tool developed following existing literature on extremism and violence, and has not been issued following a study | | 16 constructs (yellow, orange, red)  4-point scale (0 = no record/not known, 1 = low evidence, 2 = medium evidence, 3 = good evidence) |
| IVPG (study B) | | U.K.  Individuals convicted of offences involving violent extremism | Violent extremism | N = 182  49% terrorists (90)  18% school shooters (33)  11% animal rights activists (20)  10% Irish Republican Army activists (18)  9% far-right activists (17)  3% violent Sikh militants (4) | Range, M and SD not stated | 16 constructs (yellow, orange, red)  4-point scale (0 = no record/not known, 1 = low evidence, 2 = medium evidence, 3 = good evidence) |
| **Tools developed as research measures** | | | | | |  |
| 1992-RWA | | Canada  Parents of college students at Wilfred Laurier University | Right-wing authoritarianism | N = 491  50% male (244)  50% female (247) | 36 to 77  M = 47.3  SD not stated | 30 (authoritarian submission, authoritarian aggression, conventionalism)  9-point Likert scale |
| RF-R | | Canada  Students at the University of Manitoba and their parents | Religious fundamentalism | N = 151  N_students_ = 1,235  N_parents_ = 502  Gender distribution not stated | Range, M and SD not stated | 12 (no subdivision indicated)  8-point Likert scale |
| PHS | | Israel  Incarcerated Palestinian and Israeli Jewish terrorists, and Palestinian and Israeli controls | Pathological hatred | N = 151  N_terrorists_ = 90  N_controls_ = 61  Gender distribution not stated | Range, M and SD not stated | 142 (ethnocentrism, authoritarianism and anti-humanism, anti-perspectivism, general fundamentalism, exclusionary fundamentalism, biophilic vs necrophilia attitudes, gender roles attitudes, intolerance and ambiguity)  Specific distribution of items not accessible |
| MMPI-2 | | Israel  Incarcerated Palestinian and Israeli Jewish terrorists, and Palestinian and Israeli controls | Psychological orientations of terrorists | N = 151  N_terrorists_ = 90  N_controls_ = 61  Gender distribution not stated | Range, M and SD not stated | 567 (hypochondriasis, depression, hysteria, psychopathic deviate, masculinity-femininity, paranoia, psychasthenia, schizophrenia, hypomania, social introversion)  Dichotomous true-false scale |
| RWA-R | | Italy  Employed adults | Right-wing authoritarianism | N = 201  44% male (89)  56% female (112) | 18 to 56  M = 34.21  SD = 9.76 | 14 (7 authoritarian aggression and submission, 7 conservatism) |
| ITFS | | U.S.A.  Christians and Muslims in an African American Mosque | Intra-textual fundamentalism | N = 17  82% male (14)  18% female (3) | Range not stated  M = 49.9  SD = 12.8 | 12 (4 divine/inerrant, 2 self-interpretative, 2 privileged, 2 authoritative, 2 unchanging)  9-point Likert scale |
| ARIS | | U.S.A.  Adults | Past and future activism and radicalism intention | N = 429  52% male (223)  48% female (206) | 18 to 92  M = 46  SD = 17 | 14 (4 future activism, 4 future radicalism, 4 past activism, 2 past radicalism)  4-point Likert scale |
| NBMASA | | Pakistan  Postgraduate students of Muslim faith | Normative beliefs about anti-Semitic aggression | N = 144  44% male (64)  56% female (80) | 18 to 21  M = 21.5  SD = .48 | 6 (no subdivision indicated)  5-point Likert scale |
| MEMS | | Belarus, Chile, China, Eastern Europe, Guatemala, Korea, Malaysia, Serbia, Slovakia, U.S.A.  College students | Militant extremist mind-set | N = 2,424  36% male (869)  63% female (1,516)  1% not stated (39) | 17 to 24  M = 21  SD = 3.20 | 24 (10 proviolence, 6 vile world, 8 divine power)  5-point Likert scale |
| MDFI | | Mexico, U.S.A.  Universities, religious study groups, religious seminaries | Fundamentalism | N_Mex_ = 458  74% male (340)  26% female (118)  N_USA_ = 448  38% male (196)  61% female (273)  1% not stated (6) | Range not stated  M_Mex_ = 27.21  SD_Mex_ = 11.48  M_USA_ = 19.94  SD_USA_ = 4.27 | 56 generated questions (8 protection of revealed traditions versus rational criticism, 8 heteronomy versus autonomy and relativism, 8 traditionalism versus progressive religious change, 8 sacralisation versus secularisation of the public arena, 8 secular culture perceived as a threat versus secular culture embraced, 8 pluralism versus religious centrism, 8 Millennial-Messianic imminence versus prophetic scepticism)  4-point Likert scale  15 final items identified (5 external versus internal authority, 5 fixed versus malleable religion, 5 worldly rejection versus worldly affirmation)  4-point Likert scale |
| RF-I | | Italy  Volunteers in local churches or places of worship | Religious fundamentalism | N = 250  42% male (106)  58% female (144)  N_1_ = 125  44% male (55)  56% female (70)  N_2_ = 125  41% male (51)  59% female (74) | 20 to 65  M_1_ = 40  SD_1_ = 14.8  M_2_ = 42  SD_2_ = 14.1 | 9 (belief, scepticism)  5-point Likert scale |
| SyfoR | | East London, Bradford, U.K.  Pakistani and Bangladeshi Muslims | Sympathies for radicalisation | N = 608  56% male (341)  44% female (267) | 18 to 45  M and SD not stated | 16 (no subdivision indicated)  7-point Likert scale |
| IFS | | Jakarta, Depok, Bogor, Bekasi, Tangerang, Indonesia  High school and university Muslim students | Islamic fundamentalism | N = 309  45% male (140)  55% female (169) | 14 to 29  M = 17.85  SD not stated | 9 (no subdivision indicated)  + 3 items (support for Acts of Terrorism Scale)  6-point Likert scale |
| SSS (study A) | | No specified country  Via amazon.com’s Mechanical Turk (MTurk) online survey programme | Readiness to self-sacrifice and willingness to engage in extreme behaviours | N = 76  63% male (48)  37% female (28) | Range not stated  M = 31  SD = 11.77 | 10 (no subdivision indicated)  7-point Likert scale  5 additional questions measuring willingness to engage in extreme means (7-point Likert scale)  8 additional questions measuring attitudes toward individuals who do not respect the environment (7-point Likert scale) |
| SSS (study B) | | Boosa, Sri Lanka  Incarcerated individuals associated with the LTTE (Liberation Tigers of Tamil Eelam) | Readiness to self-sacrifice and willingness to engage in extreme behaviours | N = 234  100% male (234) | Range not stated  M = 32.7  SD = 6.4 | 10 (no subdivision indicated)  7-point Likert scale  2 additional questions measuring emotional hostility (5-point Likert scale)  3 additional questions measuring dissipation-rumination (7-point Likert scale) |
| ARIS-S | | Spain  Sympathizers and activists in the 15-M anti-austerity movement | Activism and radicalism intention | N = 133  44% male (58)  56% female (75) | Range not stated  M = 29.05  SD = 9.16 | 8 items (4 future activism, 4 future radicalism) |
| Schbley (2003) | | Beirut, Lebanon  Hizbullah’s Istishhadeens militants participating in a military exhibition parade | Psychological profile of religious terrorists | N = 341  Gender distribution not stated (majority of males) | 22 to 27  M and SD not stated | 50 questions (terrorism, martyrdom, Marjah’s commitment level, ethno-religious identity, religious training, theocracy, violence in the name of Allah, dogma-induced psychotic depression, schizophrenia criteria by religious leadership, absolutism, geo-cultural mobility, employability, terrorism directed at government decisions)  4-point Likert scale  32 final traits identified |
| TCS | | U.S.A.  Islamic insiders (critics, defenders, mainstream Muslims) | Characteristics of sleeper cell terrorists | N = 33  69% male (23)  31% female (10) | 17 to 62  M = 33.2  SD not stated | 62 risk factors (4 connection with foreign countries, 10 preparations, 11 attitudes, 5 identity, 6 social activities, 7 miscellaneous, 4 background, 15 controls)  5-point scale (totally irrelevant, possibly relevant, suspicion aroused, strong concern, investigation advisable) |
| TRAP-18 | | U.S.A., Europe  Lone-actor terrorists | Terrorist radicalisation | N = 111  Gender distribution not stated | Range, M and SD not stated | 18 indicators (8 proximal warning behaviours, 10 distal characteristics for active risk management or active monitoring) |
| **Inventories not generated from a study** | | | | | | |
| Ross (1994) | | U.S.A. | Psychological causes of terrorism | NA | NA | 9 psychological traits and processes acquired (no subdivision indicated) |
| Vaisman-Tzachor (2006) | | U.S.A. | Psychological makeup of terrorists | NA | NA | 5 traits (no subdivision indicated) |
| Horgan (2008) | | U.S.A. | Involvement in terrorism | NA | NA | 6 predisposing risk factors (no subdivision indicated) |
| Saucier et al. (2009) | | Europe, Middle East, Africa, South Asia, East Asia, Latin America, North America | Militant extremism mind-set | NA | NA | 16 final key themes identified (no subdivision indicated) |
| Kebbell and Porter (2012) | | Australia  Convicted terrorists | Violent extremism against the West | NA | NA | 21 risk factors identified (6 standard, 5 moderate, 6 higher, 4 extreme) |
| Monahan (2012) | | U.S.A. | Terrorism | NA | NA | 4 final categories of variables identifies (no subdivision indicated) |
| USAID (2013) | | Kyrgyzstan | Engagement in violent extremism or insurgency | NA | NA | 14 drivers identified (5 individual-level, 2 group-level, 7 political- and societal-level) |
| Borum (2014) | | U.S.A. | Involvement in violent extremism | NA | NA | 3 psychological vulnerabilities, 5 attitudinal propensities |
| EMI-20 | | Europe | Dangerous trends in individuals driven by ideological extremism | NA | NA | 20 indicators (no subdivision indicated) |

NA, not applicable
